# Supplementary material for: Graphene oxide enabled long-term enzymatic transesterification in an anhydrous gas flux
Source: Nat Commun. 2019 Jun 18;10:2684. doi: 10.1038/s41467-019-10686-z (PMC6582274; doi:10.1038/s41467-019-10686-z)
Supplement: Supplementary file 1 — Supplementary information [file 41467_2019_10686_MOESM1_ESM.pdf]

Supplementary Information

Graphene Oxide Enabled Long-Term Enzymatic Transesterification in an Anhydrous Gas Flux

Xu et al.

METHODS

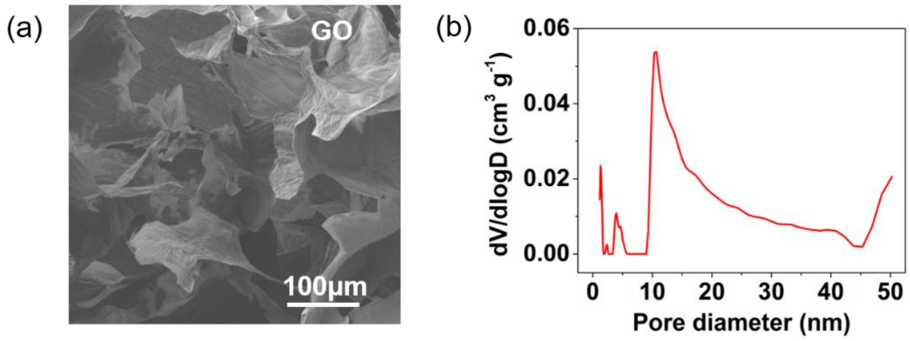

Supplementary Figure 1. Characterization of GO and LGA. (a) The structure of freeze-dried GO; (b) the BET results of LGA. Source data are provided as a Source Data file.

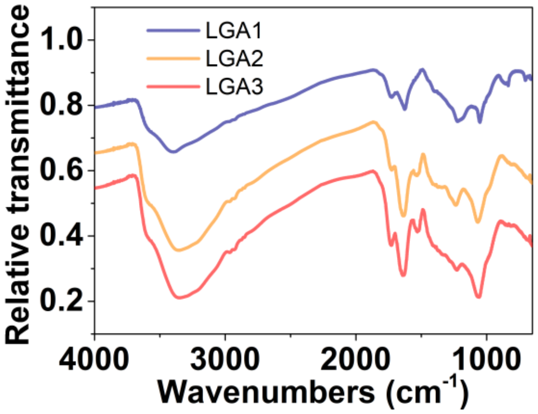

Supplementary Figure 2. FTIR spectra of LGA1, LGA2 and LGA3. Source data are provided as a Source Data file.

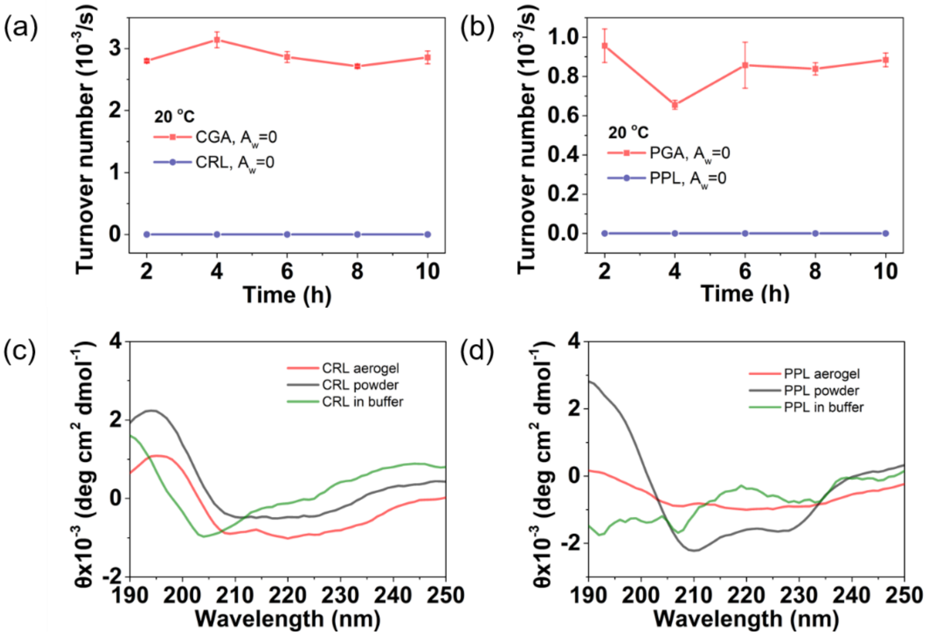

Supplementary Figure 3. Enzyme performance and structure characterization of CRL and PPL in aerogels. (a) Turnover numbers of CGA and CRL at 20 ºC; (b) turnover numbers of PGA and PPL at 20 ºC; (c) solid-state circular dichroism spectra of CGA, CRL powder and CRL in a phosphate buffer; (d) solid-state circular dichroism spectra of PGA, PPL powder and PPL in a phosphate buffer. Error bars represent the s.d. of two replicates. Source data are provided as a Source Data file.

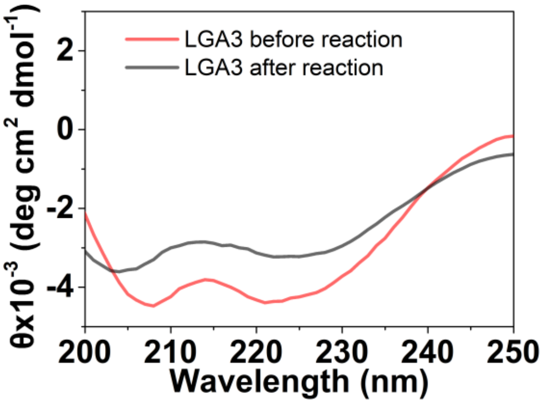

Supplementary Figure 4. Solid-state circular dichroism spectra of LGA3 before and after 10-hour reaction at A_W=0.34 and 80 ºC. Source data are provided as a Source Data file.

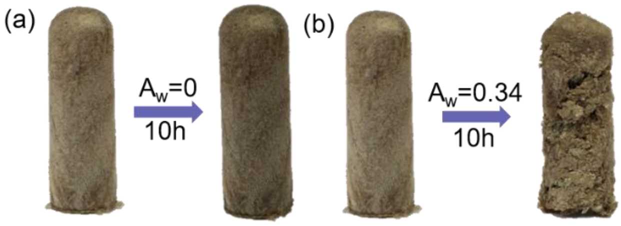

Figure 5. Morphological changes of LGA3 at (a) A_w=0 and (b) A_w=0.34.
